# An in‐depth analysis of the immunomodulatory mechanisms of intervertebral disc degeneration

**DOI:** 10.1002/jsp2.1233

**Published:** 2022-12-08

**Authors:** Chao Song, Weiye Cai, Fei Liu, Kang Cheng, Daru Guo, Zongchao Liu

**Affiliations:** ^1^ Department of Orthopedics and Traumatology (Trauma and Bonesetting) The Affiliated Hospital of Traditional Chinese Medicine of Southwest Medical University Luzhou China

**Keywords:** cell death mode, immune regulation, intervertebral disc degeneration, signaling pathway

## Abstract

Intervertebral disc degeneration (IVDD) is the pathological basis of disc herniation, spinal stenosis, and other related diseases, and the lower back pain it produces lays a heavy financial burden on individuals and society. Thus, it is essential to comprehend IVDD's pathophysiology. Numerous factors, such as inflammatory factors, oxidative stress, apoptosis, matrix metalloproteinases, are linked to IVDD pathogenesis. Despite the fact that many researches has provided explanations for the pathophysiology of IVDD, these studies are typically singular, restricted, and isolated, expound only on one or two components, and do not systematically analyze and summarize the numerous influencing elements. In addition, we discovered that the incidence of many chronic diseases in the field of orthopedics may be thoroughly and systematically defined in terms of immunological systems. In order to provide a theoretical foundation for an in‐depth understanding of the pathological process of IVDD and the formulation of more effective prevention and treatment measures, this review provides a comprehensive and systematic account of the pathogenesis of IVDD from the physical to the molecular barriers of the intervertebral disc, from the nucleus pulposus tissue to the cellular to the immune‐molecular level.

## 1 INTRODUCTION

Intervertebral disc degeneration (IVDD) is one of the common causes of low back pain in middle‐aged and elderly people, and lower back pain affects around 40% of the world's population, hurting not only patients' quality of life but also society and the healthcare system.^1^, ^2^ The intervertebral disc, the largest area of nonvascular tissue in the body, is primarily made up of the medullary nucleus pulposus (NP) tissue in the center, annulus fibrosus (AF) on either side, and upper and lower cartilage endplates (CEP).^3^ IVDD mainly leads to lumbar disc herniation (LDH) and lumbar disc degeneration (LDD).^4^ The pathogenesis of IVDD may involve a mechanical injury that ruptures the intervertebral disc's annulus fibrosus partially or completely, the outward protrusion of the NP compresses the nerve root and results in a variety of symptoms, or a combination of factors that lead to the intervertebral disc structure's degeneration and the development of an inflammatory response.^5^

At the moment, there are three main explanations for the pathophysiology of IVDD: (1) mechanical compression theory, (2) chemical radiculitis theory, (3) autoimmune theory.^6^ Mixter and Baar published the first case of LDH with sciatica in 1934, claiming that LDH was caused primarily by mechanical injury.^7^ Mechanical compression of the nerve roots by the NP tissue, according to the theory, causes congestion and edema in the nerve roots and surrounding tissues of the corresponding segment, eventually leading to symptoms such as low back pain and sciatica.^8^ However, this theory was challenged by other researchers, and Kawakami et al. discovered that in some LDH patients, the severity of pain was unrelated to the degree of nerve compression,^9^ Desmoulin et al. also concluded from observing cases that symptoms in patients with herniated discs are caused by the bulging NP pulling on the nerve roots, rather than just mechanical compression.^10^ Therefore, it is insufficient to depend exclusively on the mechanical compression explanation to describe the pathophysiology of IVDD. Many scholars now agree that mechanical compression hypothesis can only partially explain the macroscopic etiology of IVDD and that the pathogenesis of IVDD must be addressed at the cellular‐molecular level. Kang JD et al. discovered in 1995 that a herniated intervertebral disc can produce matrix metalloproteinases (MMPs), nitric oxide (NO), and inflammatory mediators such as interleukin 6 (IL‐6) and prostaglandin E2 (PGE2) through tests,^11^ Burke et al. clearly proposed in 2002 that herniated intervertebral disc tissue produces proinflammatory mediators and cytokines,^12^ and the chemical radiculitis theory was born. From a microscopic perspective, the chemical radiculitis theory largely explains the pathophysiology of IVDD,^13^ which believes that inflammatory factors are the core of pain and spinal degeneration.^14^ As a result, it has been proposed that the lower back pain associated with IVDD is caused by nerve radiculitis, which is triggered by biologically active chemicals that regulate autoimmunity in the body.^15^

Inflammatory and immunological responses are interconnected and interact with one another, as demonstrated by several research. Starkweet et al. discovered that herniated NP tissue or disc fragments in the epidural space activated the neuroimmune system,^16^ inducing the release of a high number of inflammatory cells, which are macrophages, T cells, and a few monocytes from the degenerating disc.^17^ When these inflammatory cells get activated, they release cytokines, collagenase, and other lytic enzymes, which contribute to radiculitis and low back pain.^18^ Wang et al. also discovered that when the human immune system is exposed to intervertebral disc tissue, macrophage infiltration and the release of IL‐6, interleukin 1β (IL‐1β), and TN (TNF) are linked to the subsequent autoimmune reaction.^17^ Djuric N et al. discovered that co‐culture of NP cells and macrophages in degenerative intervertebral discs can release inflammatory factors such as IL‐6, IL‐8, TNF, and NO, which can be inhibited by the betamethasone medication.^19^ In addition, monocyte chemoattractant protein 1 (MCP‐1) released from the intervertebral disc can also amplify macrophage infiltration and inflammatory responses, it can be seen that the immune response is involved in the pathogenesis of IVDD disease.^20^

## 2 THE AUTOIMMUNE THEORY OF IVDD

Bobechko proposed the autoimmune theory of LDH in 1965, implying that the pathological process of IVDD was mediated by activation of the body's immune system, but the specific mechanism was not thoroughly investigated because the cellular molecular study of IVDD was not well developed at the time.^21^ In‐depth research on the anatomy of the intervertebral disc has revealed that the NP is adequately shielded from the humoral environment by the semi‐permeable membrane of the disc.^22^ Due to the closed construction, the immune system is unable to recognize the NP tissue within the disc, preventing potentially damaging immunological reactions.^22^ Studies have also discovered that several cytokines in IVDD, including Fas Ligand (FasL), which can function as a molecular barrier by preventing vascular infiltration and immune cell recruitment, block the infiltration of inflammatory substances and immune cells^23^; Wiet et al. found that the conditioned medium from healthy annulus fibrosus inhibited mast cell activation by downregulating the expression of vascular endothelial growth factor (VEGF), tumor necrosis factor α (TNF‐α), interleukin 1 (IL‐1), and chemokine (C—C motif) ligand 2 (CCL2/MCP‐1), and inhibit mast cell induced angiogenesis.^24^ It is clear that these immunosuppressive molecules, along with annulus fibrosus and cartilage endplates, constitute a blood–nucleus–barrier (BNB) that separates myeloid tissue from bodily fluids and reduces the body's inflammatory immune response.^3^ When the BNB is destroyed, the disc's structures trigger an immune inflammatory cascade. NP tissue is regarded by the immune system as a “antigen,” and antibodies made by immune cells bind to it to form an antigen–antibody complex.^25^ Through a subsequent cascade reaction, this antigen–antibody complex eventually results in symptoms like back pain in IVDD patients.^3^, ^25^ The above concept was also confirmed by Satoh et al., who found that antigen–antibody complexes exist in prominent NP tissues,^26^ which can activate the expression of various immune cells.

**FIGURE 1:**
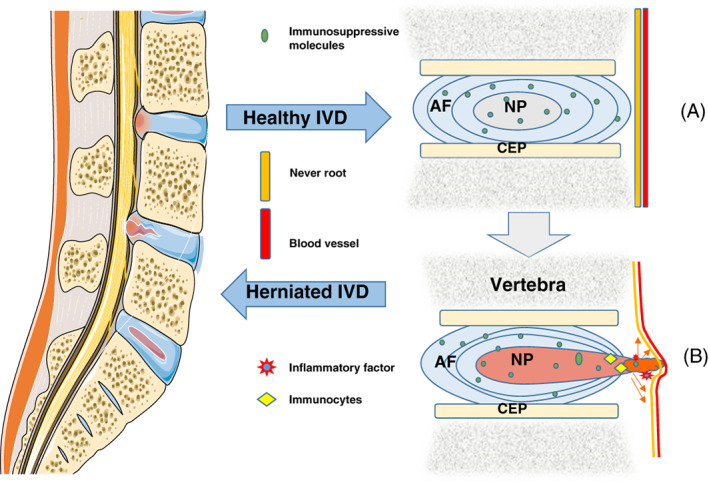
Schematic representation of the immune barrier of the intervertebral disc. (A). In a normal intervertebral disc, the blood–nucleus–barrier (BNB) consists of annulus fibrosus (AF), cartilage endplate (CEP) and immunosuppressive molecular factors. BNB will isolate the central nucleus pulposus (NP) from the host's immune system, providing the basis for disc homeostasis. (B). Breakdown of BNB leads to exposure of NPs and induces an autoimmune response. This effect causes activation of immune cells and infiltration of inflammatory factors, promoting immune stress in nerve roots, leading to disc degeneration

Geiss et al. discovered that subcutaneous injection of autologous NP increased the number of activated T and B cells in the degenerating disc to determine the type of immune cells present.^27^, ^28^ On the other hand macrophages are important cells for the activation of the human immune system, which mainly play the role of immune defense, immune homeostasis, and immune surveillance. Recently, Monchaux et al. found the presence of macrophages and monocytes in herniated canine intervertebral discs.^29^ Lee et al. also degenerated intervertebral discs in mice and found macrophage infiltration and radiculitis.^30^ Furthermore, Takada et al. observed that mechanical hyperalgesia in a rat intervertebral disc model was substantially correlated with macrophage infiltration and the up‐regulation of TNF‐α, IL‐6, IL‐8, and COX‐2.^31^ These studies suggest that a variety of immune cells in the body and the factors they produce are involved in the process of IVDD, and macrophages seem to play an important role in this pathological process. In conclusion, herniated disc tissue contacts the surrounding disc, penetrates the protective barrier, and activates the immune system,^32^ mediates the production of lymphocytes, immune cells. Among them, immune cells such as macrophages, T cells and neutrophils secrete TNF‐α, IL‐1β, IL‐17, Interferon‐γ (IFN‐γ) and other inflammatory‐related factors, which lead to inflammatory response and cause intervertebral disc‐derived pain.^17^ (Figure 1).

## 3 INNATE IMMUNE RESPONSE TO IVDD

Immune response refers to the whole process of the immune system recognizing and clearing “nonself” substances, which can be divided into two categories: innate immunity and adaptive immunity.^33^ Monocytes/macrophages, neutrophils, NK cells, and other innate immune cells and chemokines, as well as TNF, interleukin, and other cytokines, make up the body's initial line of defense against infection.^34^ Adaptive immune response is the whole process in which T and B lymphocytes in vivo activate, proliferate, differentiate into effector cells and produce a series of biological effects after being stimulated by “nonself” substances.^35^ The effector molecules of adaptive immunity encourage the response of innate immunity through positive feedback, and innate immunity is a requirement and beginning element of adaptive immunity.^34^, ^35^

The prominent NP tissues during IVDD first activate innate immune cells, secrete cytokines, regulate immune responses and mediate inflammation. It was found that the levels of monocytes/macrophages, eosinophils and basophils were significantly increased in patients with IVDD.^36^ Among them, monocytes are the key markers for the onset, progression and remission of IVDD.^37^ Monocytes derived from bone marrow hematopoietic stem cells are the largest blood cells and the largest white blood cells in the blood, and are an important part of the body's defense system.^38^ On the one hand, it contains multiple pattern recognition receptors that activate proinflammatory factors in the immune response, directly participate in phagocytosis, cytokinesis, or receptor‐mediated cytokinesis to ingest antigens, process them to present them to T cells for activates a series of immune responses,^13^ on the other hand, monocytes gradually transform into macrophages in the degenerated disc, thereby aggravating the T cell‐mediated inflammatory response. Furthermore, NP tissue stimulated the production of natural killer (NK) cells with surface markers CD16 and CD56, but how NK cells play a role in IVDD remains to be further investigated.^44^

**FIGURE 2:**
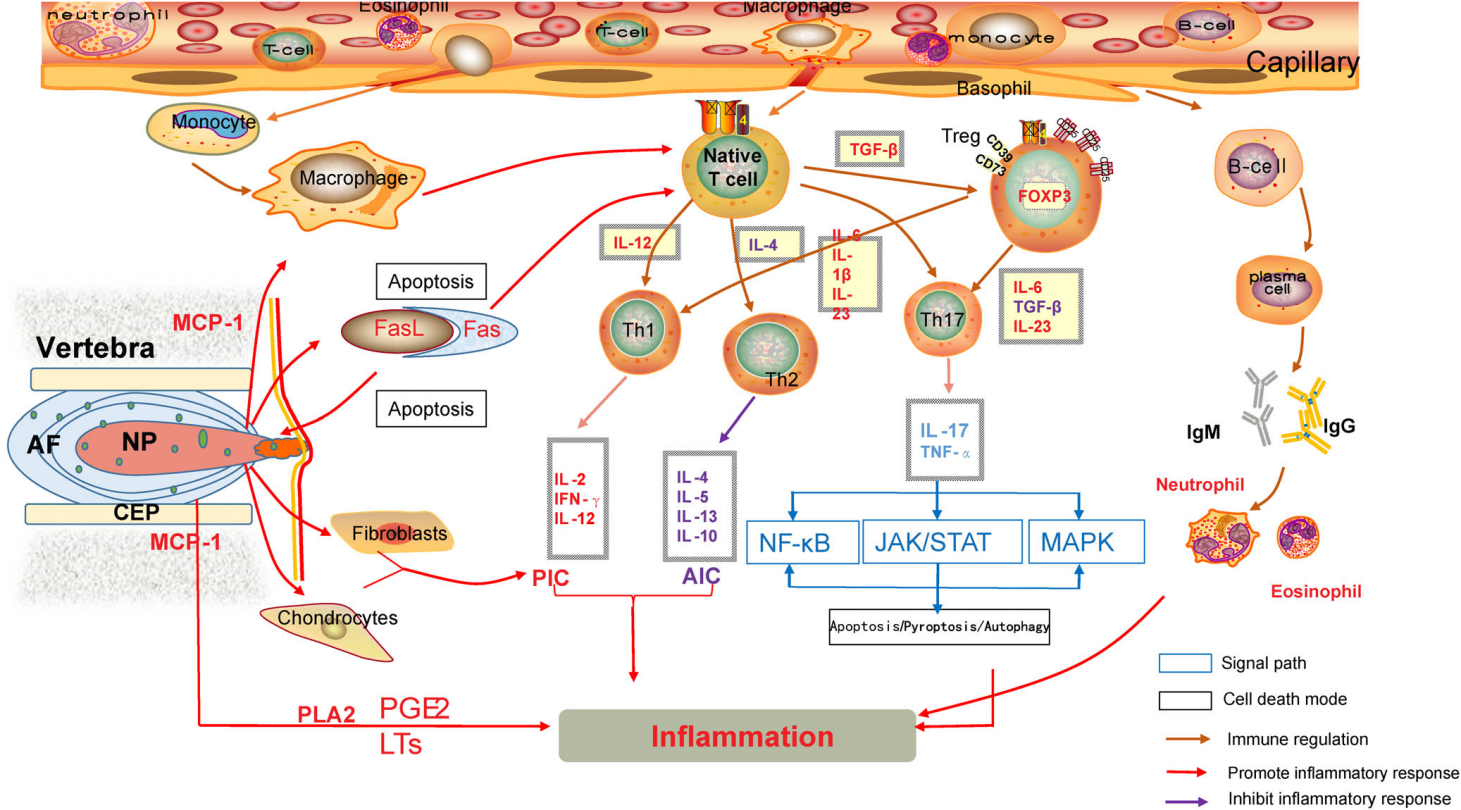
Schematic showing the immune cascade during disc‐related pain generation. When the prominent nucleus pulposus tissue breaks through the immune barrier and is recognized by the immune system, immune cells (such as T cells and macrophages) in the blood circulation are activated and released; at the same time, inflammatory mediators (TNF‐α, IFN‐γ, IL are released, etc.); and inhibitory mediators (TGF‐β, IL‐4/10, etc.). Ultimately, immune cells from the activated NPs and the blood work together to release inflammatory cytokines that activate sensory neurons and produce pain. AIC, anti‐inflammatory cytokines; MCP‐1, monocyte chemoattractant protein‐1; PIC, proinflammatory cytokines; PLA2, phospholipase A2

IVDD is controlled by cytokines from the innate immune response. In degenerative disc tissue, TNF‐α is highly expressed, which can help IL‐17 generated by Th17 mediate signaling pathways including MAPK, causing inflammation to worsen and extracellular matrix to break down. It also causes IVDD by mediating IL‐21 through the JAK–STAT pathway. During the process, there is more inflammation, which because the immune response produces a variety of inflammatory cytokines.^39^ IL‐1β regulates the body's immune signals by initiating MAPK and NF‐κB signaling pathways, causing inflammatory response and apoptosis, and plays a role in the degradation of the extracellular matrix of the intervertebral disc.^40^ Furthermore, in degenerated disc tissue, it was found that Fas and Fas ligand (FasL) are highly expressed,^41^ and FasL promotes apoptosis of vascular endothelial cells and immune cells (including macrophages and CD8+ T cells).^23^, ^42^ At the same time, it is also an inflammation‐inducing factor that can induce certain cells, including osteoclast precursors, peritoneal exudative cells, NP cells, and so on^43^ to secrete IL‐1β, IL‐18, TNF‐α, etc. Inflammatory factors directly up‐regulate the expression of various chemokines.^44^ Dunfu Han et al. also demonstrated the role of FasL in rat degenerative NP cells, namely up‐regulation of Fas expression and activation of the Fas/FasL pathway leading to apoptosis.^45^ (Figure 2).

## 4 ADAPTIVE IMMUNE RESPONSE TO IVDD

### 4.1 Th1 and Th2 mediate basal immune regulation

In degenerated intervertebral disc, the adaptive immune response includes two categories: cellular immune response and humoral immune response. The cellular immune response is triggered by the vascular endothelial cells that grow around the injured intervertebral disc. The lymphocyte aggregates migrate to the vicinity of the injured intervertebral disc, and after recognizing the “antigen,” T lymphocytes are activated. T cell‐mediated immune response is the main cellular immune response mode of IVDD,^27^ which plays an important role in the occurrence of IVDD and pain.^46^ Studies have shown that T lymphocytes are mainly differentiated into Th1, Th2, and Th17 after being activated by the prominent NP tissue.^47^, ^48^, ^49^, ^50^ Studies have found that T cell activation is a unique signal of cell‐mediated autoimmunity, and Th1 and Th2 mediate basal immune regulation in IVDD. (1) Th1 can stimulate and control macrophage expression. Through the synthesis of IFN‐γ factors and the binding of CD40L on the surface of Th1 to CD40 on the surface of macrophages, Th1 sends activation signals to macrophages. On the other hand, by increasing the expression of immune molecules and secreting cytokines, activated macrophages can likewise boost the impact of Th1. Monocytes/macrophages and lymphocytes are encouraged to adhere to vascular endothelial cells by TNF‐α and MCP‐1 produced by Th1. These cells subsequently cross the vascular wall to chemotactically go into the nearby degenerating NP tissue.^51^ (2) Th1 is a cytokine that controls the activation and proliferation of lymphocytes. The immune response is amplified by Th1 because it releases cytokines like IL‐2 and IL‐12 that can encourage the activation and proliferation of Th1, Th2, and NK cells.^52^ (3) The IFN‐γ factor secreted by Th1 can also promote B cells to produce opsonizing antibodies, further enhancing the phagocytosis of macrophages.^53^ (4) Lymphotoxin and TNF‐α produced by Th1 can activate neutrophils and promote their inflammatory response. (5) Th2 cell secretion IL‐4, IL‐5 and Cytokines such as IL‐10 and IL‐13 assist and promote the proliferation and differentiation of B cells into plasma cells and produce antibodies.^47^, ^54^ In summary, Th1 cells mainly produce PIC such as IFN‐γ and IL‐12, which are involved in inflammation and cellular immune responses, while Th2 cells produce IL‐6, IL‐13 and other anti‐inflammatory cytokines (AIC) are involved in humoral immune responses.^55^ In a healthy organism, Th2‐dependent cytokines suppress cellular immunological and inflammatory responses, but when they interact with macrophages around herniated discs, they boost the Th1 response and suppress the Th2 response, resulting in an inflammatory immune response to IVDD tissue^50^ (Figure 2).

Immune glycoprotein antibodies that mediate humoral immune responses are produced by proliferating and differentiated plasma cells of B lymphocytes, and they bind to IgG and IgM antigens in the peripheral blood and cerebrospinal fluid of patients with IVDD to mediate humoral immunity at the same time as cellular immunity.^56^, ^57^ Humoral immunity mainly leads to the accumulation of inflammatory cells and IGs, and the subsequent release of inflammatory mediators causes local inflammation and pain. Among them, IgG and IgM IGs bind to antigens, expose the Fc portion of the complement‐binding site of IGs, bind to complement C1q and activate the classical complement pathway, thereby causing an inflammatory response.^58^

### 4.2 Th17‐mediated regulation of related signaling pathways

**FIGURE 3:**
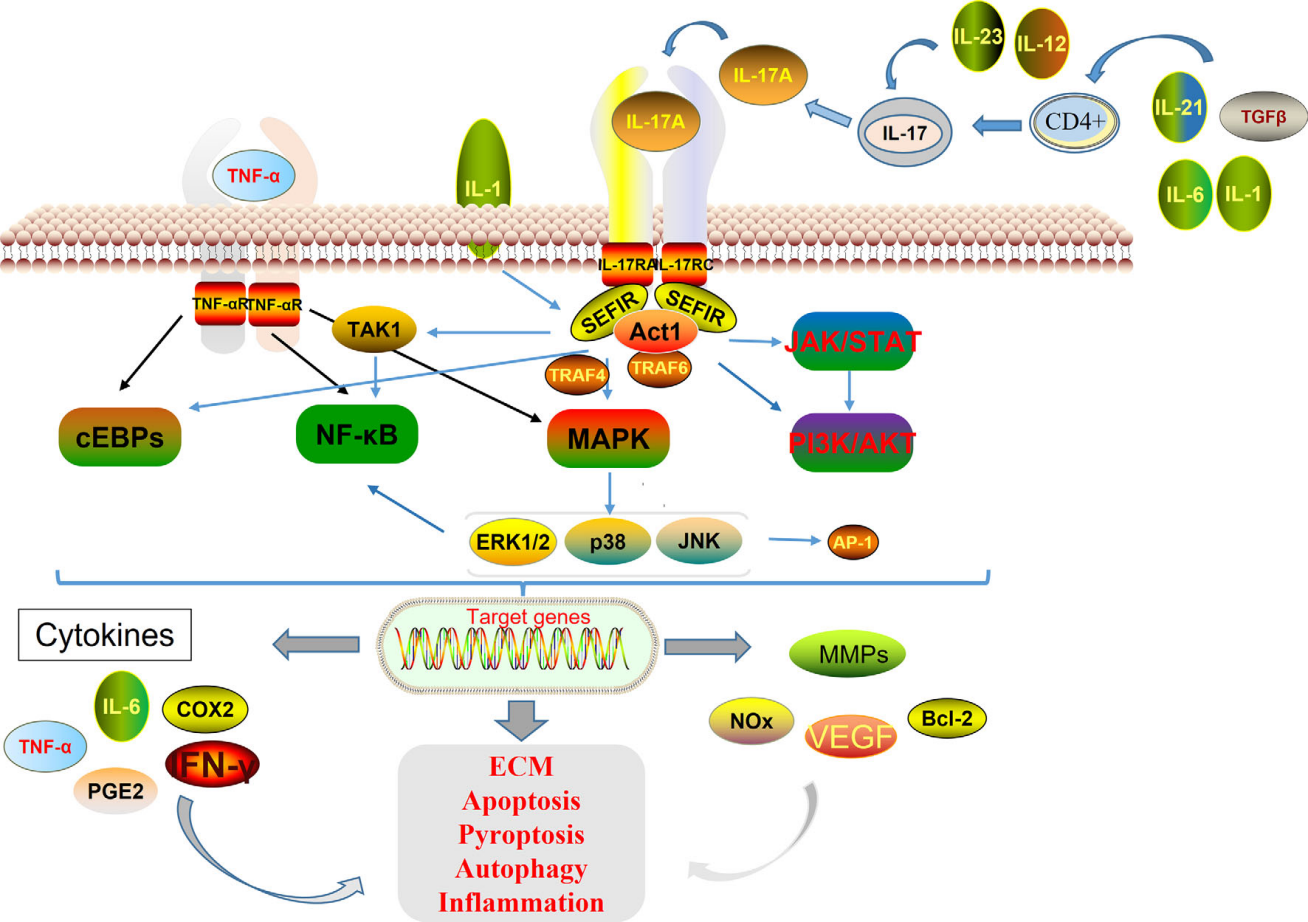
Production of IL‐17A and its receptor and transduction signaling pathways. IL‐17A is produced by Th17 cells, binds to receptors composed of IL‐17RA and IL‐17RC, and interacts with Act1. Subsequently, Act1 activates multiple independent signaling pathways. Furthermore, TNF‐α stimulates NF‐κB and MAPK pathways and C/EBPs, and is involved in the production of cytokines, chemokines, and extracellular matrix degrading factors in IVD, leading to inflammation and extracellular matrix degradation

IL‐17A, an effector released by Th17 cells, is crucial in the pathogenesis of IVDD and inflammation.^59^ According to studies, blood levels of IL‐12 and IL‐17 in people with LDH are much higher than they are in healthy controls.^60^ Numerous molecules, including IL‐12, TGF‐, IL‐6, IL‐1, IL‐21, and IL‐23, cause CD4+ Th17 cells to produce IL‐17A, which activates downstream signaling pathways that result in IVDD.^61^, ^62^ Five members of the IL‐17 receptor (IL‐17R) family, IL‐17RA through IL‐17RE, have structural characteristics with the Toll‐IL‐1R (SEFIR) domain.^63^ The IL‐17A, IL‐17RA, and IL‐17RC heterodimeric receptor complex starts the NF‐B and MAPK pathways, which are the most important downstream signaling pathways^63^, ^64^ (Figure 3).

**FIGURE 4:**
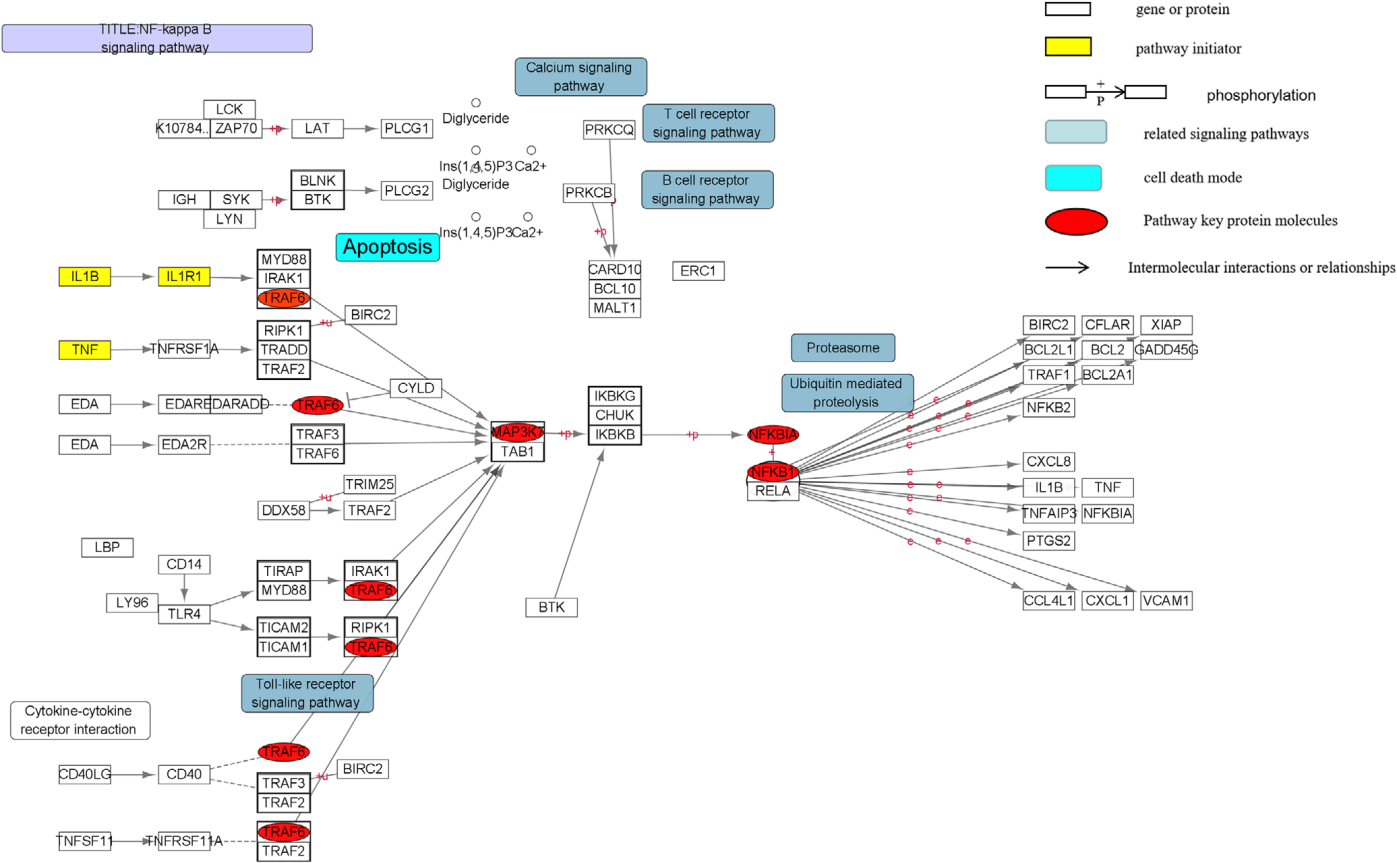
Diagram of the NF‐κB signaling pathway and its related mechanisms. The pathway map shows that IL‐1β, TNF‐α and other factors are used as initiators to mediate signal expression. TRAF6 and TAK1 are NF‐κB pathway hub factors, which ultimately lead to the expression of inflammatory factors such as IL‐18, IL‐1β, COX‐2, and MMP. Expression, leading to increased inflammation and extracellular matrix degradation(This picture is done by myself using cytoscape)

NF‐B activator 1 (ACT1), which starts the NF‐B pathway, has a SEFIR domain and a TRAF6 binding motif.^65^ When IL‐17A binds to IL‐17R (IL‐17RA/IL‐17RC), Act1 binds to IL‐17A receptor through SEFIR domain, and TRAF6 binds to Act1 binding motif. Subsequently, activated TRAF6 activates the transforming growth factor beta‐activated kinase Tak1, which activates the NF‐κB signaling pathway.^65^, ^66^, ^67^ Through KEGG signaling pathway database query and literature search, it was found that IL‐17A alone is not enough to strongly activate NF‐κB, so IL‐17A and other cytokines such as TNF‐α, IL‐1 co‐stimulate NF‐κB and enhance Stability of proinflammatory cytokine and chemokine gene expression^63^, ^68^ (Figure 4). Activation of NF‐κB signaling pathway releases a variety of inflammatory cytokines, such as TNF‐α, IL‐2, IL‐6, IFN‐γ, and catabolic enzymes, including MMP‐3, MMP‐9, MMP‐13, ADAMTS‐4, and ADAMS‐5,^69^ also reduce the expression of collagen type II alpha 1 (COL2A1) and agglutinin,^70^ thereby mediating extracellular matrix degradation. In addition, the research group previously found that NF‐κB pathway amplifies the inflammatory response by regulating the expression of NLRP3 in cells, leading to pyroptosis and the release of IL‐1β and IL‐18. Finally, it can be said that the NF‐B pathway is crucial for controlling IVDD's inflammatory response, nucleus pulposus apoptosis, and extracellular matrix dissolution.^71^, ^72^

**FIGURE 5:**
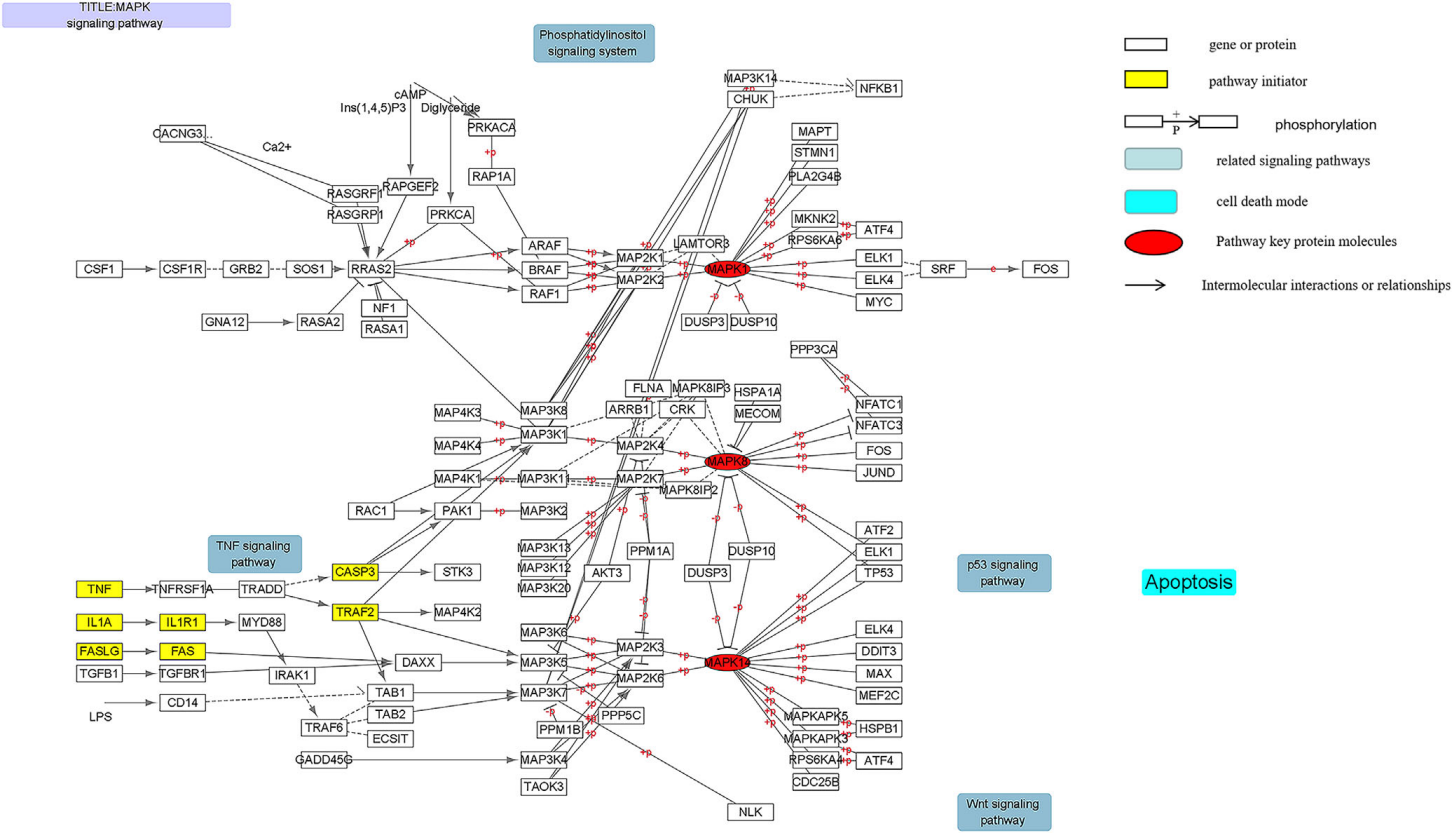
Diagram of the MAPK signaling pathway and its associated mechanisms. The pathway map shows that factors such as IL‐1 and TNF are used as the initiator to mediate the expression of signals. JNK, p38, and ERK are the three subtypes of the currently known MAPK pathway, which ultimately mediates apoptosis. In addition, FasL/Fas signaling‐mediated apoptosis is also expressed in this pathway. This pathway also crosstalks with the NF‐κB signaling pathway, and they share a common promoter (this picture is done by myself using cytoscape)

Another in vitro investigation from NP of individuals with IVDD also demonstrated the function of IL‐17A in IVDD inflammation via modulating the mitogen‐activated protein kinase (MAPK) pathway through the development of PGE2 and COX‐2.^73^, ^74^ IL‐17A stimulates the recruitment of TRAF4, which competitively binds to the same binding site of TRAF6 on Act‐1,^63^, ^75^ mediates Act1‐TRAF4, and activates the MAPK pathway^76^ (Figure 5) The MAPK pathway includes three major pathways, p38 kinase, JNK and extracellular signal‐regulated kinase (ERK), which play a crucial role in IVDD.^77^, ^78^ AP‐1 is a downstream transcription factor of the MAPK pathway, involved in the regulation of COX‐2 and IL‐6, cell proliferation, apoptosis and inflammation,^79^, ^80^, ^81^ and its regulation level is mainly concentrated in the transcription level of Jun and Fos genes. IL‐17A increases COX‐2 and PGE2 expression by activating AP‐1‐dependent p38/c‐Fos and JNK/c‐Jun signaling pathways.^73^ Studies have shown that JNK phosphorylation promotes the natural degeneration of cartilage endplate cells and reduces NP type II collagen and Aggrecan synthesis, and inhibition of JNK phosphorylation can significantly reverse this process and accelerate the proliferation of cartilage endplate cells.^82^ The above reports indicate that IL‐17A‐induced MAPK is a signaling pathway that is also involved in apoptosis, inflammatory response, and extracellular matrix degradation in the course of degenerative disc disease (Figure 3). At the same time, through the KEGG database, we found that the activation and expression of the MAPK signaling pathway is linked to the NF‐κB signaling pathway, but the specific mechanism is still unclear and needs to be further studied. Thus, the activation of Th17 and secretion of IL‐17 play an important role in IVDD.^83^ As a result, we hypothesize that IL‐17 may be one of the markers for the immune process activation associated with IVDD.^40^

Finally, IL‐17A can also activate the JAK/STAT pathway^84^ and induce the activation of PI3K/AKT.^85^ A study on IVDD showed that IL‐17A upregulates VEGF expression in NPs through the JAK/STAT pathway^86^ Promote the ingrowth of blood vessels in intervertebral disc lesions, thereby mediating immune and inflammatory responses.^87^ On the other hand, IL‐17A inhibits autophagy in NP cells by activating the PI3K.^88^ Autophagy has been shown to be a protective mechanism of IDD, therefore, inhibition of autophagy by IL‐17A‐PI3K/AKT activation may exacerbate disc degeneration.^89^, ^90^

## 5 IMMUNOGLOBULIN

A protein with antibody activity is called immunoglobulin, mostly found in plasma, but also in some body secretions, tissues, and other bodily fluids. It has been established that IgG is present in the intervertebral disc. The complement system is activated when IgG enters the epidural space as a result of intervertebral disc degeneration or nucleus pulposus, which causes an inflammatory reaction and low back pain in patients.^91^ Collagen I, II, V, and aggrecan were discovered in human IVDD sample by Capossela et al.^92^ By creating an IVDD model, Silva et al. discovered that human macrophages obstruct the remodeling of the extracellular matrix (ECM) in IVDD by suppressing the expression of aggrecan and type II collagen genes by IL‐1β.^93^

Despite the lack of specific antigens in the material produced by the normal NP, scholars have demonstrated the presence of IG and membrane attack complexes.^94^ Takennake detects IgG and C3 in inflamed disc tissue using an experimental animal model, showing that complement activation by antigen–antibody complexes drives the inflammatory response.^95^ Spiliopoulou et al also found that IgG and IgM levels in serum and cerebrospinal fluid were also higher.^57^ This suggests that IgG and IgM may be antibodies associated with disc degeneration. The mechanism may be that when the annulus fibrosus is ruptured or damaged, the NP is exposed as a self‐antigen, and the IgG and IgM produced around the intervertebral disc combine with specific antibodies to form immune complexes, inducing inflammatory cells such as neutrophils to aggregate and produce inflammation.^15^ IgG is the main antibody of the body's secondary immune response, and it is also the “main force” of anti‐infection. It can activate complement through the classical pathway, and can bind to Fc receptors on the surface of macrophages and NK cells to play an anti‐inflammatory effect.^92^, ^96^ IgM is the earliest antibody in the primary humoral immune response, and is the body's “pioneering force” in anti‐infection.^91^ IG‐mediated inflammatory mediators have the capacity to increase vascular permeability, encourage immune complexes to enter the wounded intervertebral disc, phagocytose both normal and pathological cells, and then release lysosomal proteases to break down and breakdown proteoglycans. Collagen molecules that cause the annulus fibrosus to split and rupture harm tissue, change the biomechanical condition, and result in IVDD.^91^, ^92^, ^96^

## 6 DISCUSSION

In the present and the future, IVDD will be a hot topic due to the acceleration of population aging. Although numerous studies have discussed the etiology of IVDD as the subject of research in the field of orthopedics, very few papers have thoroughly and methodically elucidated its mechanism from an immunological perspective. Therefore, understanding the pathogenesis of IVDD at the level of immune molecules will give us a theoretical foundation to fully and deeply comprehend the pathological process of IVDD and to develop more effective prevention and treatment measures. This will ultimately be helpful to the discovery of suitable drugs to provide more precise treatment for the majority of IVDD patients.

Innate conditions for NP to evade the body's immune system are provided by BNB, which is made up of immunosuppressive molecules, AF, and CEP.^92^ As a result of collagen in IVDD breaking through BNB and acting as an autoantigen in conjunction with nearby stimulated blood vessels and neurons, the immune system of the body is activated, leading to symptoms like low back pain.^32^ According to immunohistology, immunohistochemistry and molecular biology, the degenerated NP tissue of IVDD patients is characterized by neovascularization and the production of immune‐related inflammatory cells (monocytes/macrophages, granulocytes, T lymphocytes, and B lymphocytes) as a major feature, these cells have the ability to produce and release inflammatory mediators.^19^, ^97^ These inflammatory mediators can indirectly cause sciatica by directly stimulating nerve roots or initiating PIC and chemokines.^98^ The review found that the expression of various immune cells, signaling pathways, and cell death methods are involved in the degenerated intervertebral disc tissue, and the crosstalk between them constitutes the immune regulation mechanism of IVDD.

In conclusion, immune cells, cell death mechanisms, communication pathways, inflammatory reactions, and extracellular matrix degradation all contribute to the development of IVDD. Due to the complexity and sophistication of the human body, more work needs to be done to improve the precise immune control. Along with the crucial involvement of humoral immunity and other immune cells in the progression of IVDD, it is also vital to consider whether there are additional cell death mechanisms in degenerating intervertebral discs, as well as any distinctions between these cell death mechanisms. We need to investigate and confirm this set of issues further. Our research team will continue to investigate and substantiate the regulation of related mechanisms because it is anticipated that the regulation of intervertebral disc immunity will be a hot topic in future research.

## AUTHOR CONTRIBUTIONS

Zongchao Liu conceived the idea of the article and revised the manuscript, Chao Song organized and wrote the article, Weiye Cai, Fei Liu, Kang Cheng, Daru Guo participated in the revision and data collection of the article.

## FUNDING INFORMATION

This study was funded by the key research and development project of the Sichuan Provincial Science and Technology Program (Study on the mechanism of Duhuo Jishen decoction in inhibiting pyroptosis of nucleus pulposus cells 22ZDYF0512) and Natural Science Key Project of Sichuan Provincial Education Department (The role of SDF‐1/CXCR4 signaling axis on the extracellular matrix of degenerated intervertebral disc and its mechanism 17ZA0432).

## CONFLICT OF INTEREST

The authors have no financial or proprietary interests in any material discussed in this article.

## References

[1] Siccoli A , Staartjes VE , De Wispelaere MP , Vergroesen PA , Schröder ML . Tandem disc herniation of the lumbar and cervical spine: case series and review of the epidemiological, pathophysiological and genetic literature. Cureus. 2019;11(2):e4081. 31019859 10.7759/cureus.4081 PMC6467429

[2] Hartvigsen J , Hancock MJ , Kongsted A , et al. What low back pain is and why we need to pay attention. Lancet. 2018;391(10137):2356‐2367. 29573870 10.1016/S0140-6736(18)30480-X

[3] Sun Z , Liu B , Luo ZJ . The immune privilege of the intervertebral disc: implications for intervertebral disc degeneration treatment. Int J Med Sci. 2020;17(5):685‐692. 32210719 10.7150/ijms.42238 PMC7085207

[4] Kos N , Gradisnik L , Velnar T . A brief review of the degenerative intervertebral disc disease. Med Arch. 2019;73(6):421‐424. 32082013 10.5455/medarh.2019.73.421-424 PMC7007629

[5] Dower A , Davies MA , Ghahreman A . Pathologic basis of lumbar radicular pain. World Neurosurg. 2019;128:114‐121. 31028982 10.1016/j.wneu.2019.04.147

[6] Delgado‐López PD , Rodríguez‐Salazar A , Martín‐Alonso J , Martín‐Velasco V . Lumbar disc herniation: natural history, role of physical examination, timing of surgery, treatment options and conflicts of interests. Neurocirugia (Astur). 2017;28(3):124‐134. 28130015 10.1016/j.neucir.2016.11.004

[7] Mixter WJ . Rupture of the lumbar intervertebral disk: an etiologic factor for so‐called “sciatic” pain. Ann Surg. 1937;106(4):777‐787. 17857078 10.1097/00000658-193710000-00027 PMC1390630

[8] Blamoutier A . Nerve root compression by lumbar disc herniation: a french discovery? Orthop Traumatol Surg Res. 2019;105(2):335‐338. 30799172 10.1016/j.otsr.2018.10.025

[9] Kawakami M , Tamaki T , Hashizume H , Weinstein JN , Meller ST . The role of phospholipase A2 and nitric oxide in pain‐related behavior produced by an allograft of intervertebral disc material to the sciatic nerve of the rat. Spine (Phila Pa 1976). 1997;22(10):1074‐1079. 9160464 10.1097/00007632-199705150-00004

[10] Desmoulin GT , Pradhan V , Milner TE . Mechanical aspects of intervertebral disc injury and implications on biomechanics. Spine (Phila Pa 1976). 2020;45(8):E457‐e464. 31651681 10.1097/BRS.0000000000003291

[11] Kang JD , Georgescu HI , McIntyre‐Larkin L , Stefanovic‐Racic M , Evans CH . Herniated cervical intervertebral discs spontaneously produce matrix metalloproteinases, nitric oxide, interleukin‐6, and prostaglandin E2. Spine (Phila Pa 1976). 1995;20(22):2373‐2378. 8578386 10.1097/00007632-199511001-00001

[12] Burke JG , Watson RW , McCormack D , Dowling FE , Walsh MG , Fitzpatrick JM . Intervertebral discs which cause low back pain secrete high levels of proinflammatory mediators. J Bone Joint Surg Br. 2002;84(2):196‐201. 11924650 10.1302/0301-620x.84b2.12511

[13] Cunha C , Silva AJ , Pereira P , Vaz R , Gonçalves RM , Barbosa MA . The inflammatory response in the regression of lumbar disc herniation. Arthritis Res Ther. 2018;20(1):251. 30400975 10.1186/s13075-018-1743-4 PMC6235196

[14] Navone SE , Marfia G , Giannoni A , et al. Inflammatory mediators and signalling pathways controlling intervertebral disc degeneration. Histol Histopathol. 2017;32(6):523‐542. 27848245 10.14670/HH-11-846

[15] Cosamalón‐Gan I , Cosamalón‐Gan T , Mattos‐Piaggio G , Villar‐Suárez V , García‐Cosamalón J , Vega‐Álvarez JA . Inflammation in the intervertebral disc herniation. Neurocirugia (Astur: Engl Ed). 2021;32(1):21‐35. 32169419 10.1016/j.neucir.2020.01.001

[16] Starkweather A , Witek‐Janusek L , Mathews HL . Neural‐immune interactions: implications for pain management in patients with low‐back pain and sciatica. Biol Res Nurs. 2005;6(3):196‐206. 15583360 10.1177/1099800404272221

[17] Wang L , He T , Liu J , et al. Revealing the immune infiltration landscape and identifying diagnostic biomarkers for lumbar disc herniation. Front Immunol. 2021;12:666355. 34122424 10.3389/fimmu.2021.666355 PMC8190407

[18] Miyagi M , Uchida K , Takano S , et al. Role of CD14‐positive cells in inflammatory cytokine and pain‐related molecule expression in human degenerated intervertebral discs. J Orthop Res. 2021;39(8):1755‐1762. 32856747 10.1002/jor.24839

[19] Djuric N , Lafeber GCM , Vleggeert‐Lankamp CLA . The contradictory effect of macrophage‐related cytokine expression in lumbar disc herniations: a systematic review. Eur Spine J. 2020;29(7):1649‐1659. 31768840 10.1007/s00586-019-06220-w

[20] Risbud MV , Shapiro IM . Role of cytokines in intervertebral disc degeneration: pain and disc content. Nat Rev Rheumatol. 2014;10(1):44‐56. 24166242 10.1038/nrrheum.2013.160 PMC4151534

[21] Bobechko WP , Hirsch C . Auto‐immune response to nucleus pulposus in the rabbit. J Bone Joint Surg Br. 1965;47:574‐580. 14341081

[22] Fournier DE , Kiser PK , Shoemaker JK , Battié MC , Séguin CA . Vascularization of the human intervertebral disc: a scoping review. JOR Spine. 2020;3(4):e1123. 33392458 10.1002/jsp2.1123 PMC7770199

[23] Liu ZH , Sun Z , Wang HQ , et al. FasL expression on human nucleus pulposus cells contributes to the immune privilege of intervertebral disc by interacting with immunocytes. Int J Med Sci. 2013;10(8):1053‐1060. 23801893 10.7150/ijms.6223 PMC3691805

[24] Wiet MG , Piscioneri A , Khan SN , Ballinger MN , Hoyland JA , Purmessur D . Mast cell‐intervertebral disc cell interactions regulate inflammation, catabolism and angiogenesis in discogenic back pain. Sci Rep. 2017;7(1):12492. 28970490 10.1038/s41598-017-12666-z PMC5624870

[25] Lawson LY , Harfe BD . Developmental mechanisms of intervertebral disc and vertebral column formation. Wiley Interdiscip Rev Dev Biol. 2017;6(6):e283. 10.1002/wdev.283 28719048

[26] Satoh K , Konno S , Nishiyama K , Olmarker K , Kikuchi S . Presence and distribution of antigen‐antibody complexes in the herniated nucleus pulposus. Spine (Phila Pa 1976). 1999;24(19):1980‐1984. 10528371 10.1097/00007632-199910010-00003

[27] Geiss A , Larsson K , Rydevik B , Takahashi I , Olmarker K . Autoimmune properties of nucleus pulposus: an experimental study in pigs. Spine (Phila Pa 1976). 2007;32(2):168‐173. 17224810 10.1097/01.brs.0000251651.61844.2d

[28] Geiss A , Larsson K , Junevik K , Rydevik B , Olmarker K . Autologous nucleus pulposus primes T cells to develop into interleukin‐4‐producing effector cells: an experimental study on the autoimmune properties of nucleus pulposus. J Orthop Res. 2009;27(1):97‐103. 18634006 10.1002/jor.20691

[29] Monchaux M , Forterre S , Spreng D , Karol A , Forterre F , Wuertz‐Kozak K . Inflammatory processes associated with canine intervertebral disc herniation. Front Immunol. 2017;8:1681. 29255462 10.3389/fimmu.2017.01681 PMC5723024

[30] Lee S , Millecamps M , Foster DZ , Stone LS . Long‐term histological analysis of innervation and macrophage infiltration in a mouse model of intervertebral disc injury‐induced low back pain. J Orthop Res. 2020;38(6):1238‐1247. 31814143 10.1002/jor.24560

[31] Takada T , Nishida K , Maeno K , et al. Intervertebral disc and macrophage interaction induces mechanical hyperalgesia and cytokine production in a herniated disc model in rats. Arthritis Rheum. 2012;64(8):2601‐2610. 22392593 10.1002/art.34456

[32] Jin X , Wang J , Ge L , Hu Q . Identification of immune‐related biomarkers for sciatica in peripheral blood. Front Genet. 2021;12:781945. 34925462 10.3389/fgene.2021.781945 PMC8677837

[33] McComb S , Thiriot A , Akache B , Krishnan L , Stark F . Introduction to the immune system. Methods Mol Biol. 2019;2024:1‐24. 31364040 10.1007/978-1-4939-9597-4_1

[34] Kaur BP , Secord E . Innate immunity. Pediatr Clin North Am. 2019;66(5):905‐911. 31466680 10.1016/j.pcl.2019.06.011

[35] Bonilla FA , Oettgen HC . Adaptive immunity. J Allergy Clin Immunol. 2010;125(2 Suppl 2):S33‐S40. 20061006 10.1016/j.jaci.2009.09.017

[36] Palada V , Ahmed AS , Finn A , Berg S , Svensson CI , Kosek E . Characterization of neuroinflammation and periphery‐to‐CNS inflammatory cross‐talk in patients with disc herniation and degenerative disc disease. Brain Behav Immun. 2019;75:60‐71. 30248387 10.1016/j.bbi.2018.09.010

[37] Kawaguchi S , Yamashita T , Katahira G , Yokozawa H , Torigoe T , Sato N . Chemokine profile of herniated intervertebral discs infiltrated with monocytes and macrophages. Spine (Phila Pa 1976). 2002;27(14):1511‐1516. 12131709 10.1097/00007632-200207150-00006

[38] Kratofil RM , Kubes P , Deniset JF . Monocyte conversion during inflammation and injury. Arterioscler Thromb Vasc Biol. 2017;37(1):35‐42. 27765768 10.1161/ATVBAHA.116.308198

[39] Chen B , Liu Y , Zhang Y , Li J , Cheng K , Cheng L . IL‐21 is positively associated with intervertebral disc degeneration by interaction with TNF‐α through the JAK‐STAT signaling pathway. Inflammation. 2017;40(2):612‐622. 28233079 10.1007/s10753-017-0508-6

[40] Johnson ZI , Schoepflin ZR , Choi H , Shapiro IM , Risbud MV . Disc in flames: roles of TNF‐α and IL‐1β in intervertebral disc degeneration. Eur Cell Mater. 2015;30:104‐116. discussion 116–107. 26388614 10.22203/ecm.v030a08 PMC4751407

[41] Park JB , Park IC , Park SJ , Jin HO , Lee JK , Riew KD . Anti‐apoptotic effects of caspase inhibitors on rat intervertebral disc cells. J Bone Joint Surg Am. 2006;88(4):771‐779. 16595467 10.2106/JBJS.E.00762

[42] Sun Z , Wan ZY , Guo YS , Wang HQ , Luo ZJ . FasL on human nucleus pulposus cells prevents angiogenesis in the disc by inducing Fas‐mediated apoptosis of vascular endothelial cells. Int J Clin Exp Pathol. 2013;6(11):2376‐2385. 24228099 PMC3816806

[43] Park H , Jung YK , Park OJ , Lee YJ , Choi JY , Choi Y . Interaction of Fas ligand and Fas expressed on osteoclast precursors increases osteoclastogenesis. J Immunol. 2005;175(11):7193‐7201. 16301623 10.4049/jimmunol.175.11.7193

[44] O'Connell J , Houston A , Bennett MW , O'Sullivan GC , Shanahan F . Immune privilege or inflammation? Insights into the Fas ligand enigma. Nat Med. 2001;7(3):271‐274. 11231613 10.1038/85395

[45] Han D , Ding Y , Liu SL , et al. Double role of Fas ligand in the apoptosis of intervertebral disc cells in vitro. Acta Biochim Biophys Sin (Shanghai). 2009;41(11):938‐947. 19902128 10.1093/abbs/gmp087

[46] Akyol S , Hancı M . TH1 and TH2 cytokines production and NK cell level assessment in peripheral blood of patients with DDH. Indian J Surg. 2013;75(4):294‐297. 24426456 10.1007/s12262-012-0488-6 PMC3726805

[47] Yao Y , Xue H , Chen X , et al. Polarization of helper T lymphocytes maybe involved in the pathogenesis of lumbar disc herniation. Iran J Allergy Asthma Immunol. 2017;16(4):347‐357. 28865415

[48] Kämpe A , Knebel A , Carlson R , Rohn K , Tipold A . Evaluation of the involvement of Th17‐cells in the pathogenesis of canine spinal cord injury. PLoS One. 2021;16(9):e0257442. 34591917 10.1371/journal.pone.0257442 PMC8483396

[49] Shamji MF , Guha D , Paul D , Shcharinsky A . Systemic inflammatory and Th17 immune activation among patients treated for lumbar radiculopathy exceeds that of patients treated for persistent postoperative neuropathic pain. Neurosurgery. 2017;81(3):537‐544. 28591802 10.1093/neuros/nyx052

[50] Park JB , Chang H , Kim YS . The pattern of interleukin‐12 and T‐helper types 1 and 2 cytokine expression in herniated lumbar disc tissue. Spine (Phila Pa 1976). 2002;27(19):2125‐2128. 12394925 10.1097/00007632-200210010-00009

[51] Del Prete G . Human Th1 and Th2 lymphocytes: their role in the pathophysiology of atopy. Allergy. 1992;47(5):450‐455. 1485646 10.1111/j.1398-9995.1992.tb00662.x

[52] Hu Z , Zou Q , Su B . Regulation of T cell immunity by cellular metabolism. Front Med. 2018;12(4):463‐472. 30112717 10.1007/s11684-018-0668-2

[53] Rosloniec EF , Latham K , Guedez YB . Paradoxical roles of IFN‐gamma in models of Th1‐mediated autoimmunity. Arthritis Res. 2002;4(6):333‐336. 12453308 10.1186/ar432 PMC153838

[54] Silva LC , Ortigosa LC , Benard G . Anti‐TNF‐α agents in the treatment of immune‐mediated inflammatory diseases: mechanisms of action and pitfalls. Immunotherapy. 2010;2(6):817‐833. 21091114 10.2217/imt.10.67

[55] Nian Y , Minami K , Maenesono R , et al. Changes of T‐cell immunity over a lifetime. Transplantation. 2019;103(11):2227‐2233. 31107822 10.1097/TP.0000000000002786 PMC6814521

[56] Zhu LG , Chen X , Yu J , et al. Effect of removing dampness and promoting diuresis method on IgG, IgM and IL‐1beta, IL‐8 in serum of rats with autoimmunity induced by nucleus pulposus. Zhongguo Gu Shang. 2011;24(4):327‐331. 21604534

[57] Spiliopoulou I , Korovessis P , Konstantinou D , Dimitracopoulos G . IgG and IgM concentration in the prolapsed human intervertebral disc and sciatica etiology. Spine (Phila Pa 1976). 1994;19(12):1320‐1323. 8066510 10.1097/00007632-199406000-00003

[58] Laursen NS , Pedersen DV , Gytz H , et al. Functional and structural characterization of a potent C1q inhibitor targeting the classical pathway of the complement system. Front Immunol. 2020;11:1504. 32849513 10.3389/fimmu.2020.01504 PMC7396675

[59] Tan JH , Li ZP , Liu LL , Liu H , Xue JB . IL‐17 in intervertebral disc degeneration: mechanistic insights and therapeutic implications. Cell Biol Int. 2022;46(4):535‐547. 35066966 10.1002/cbin.11767

[60] Xue H , Yao Y , Wang X , et al. Interleukin‐21 is associated with the pathogenesis of lumbar disc herniation. Iran J Allergy Asthma Immunol. 2015;14(5):509‐518. 26742440

[61] Schinocca C , Rizzo C , Fasano S , et al. Role of the IL‐23/IL‐17 pathway in rheumatic diseases: an overview. Front Immunol. 2021;12:637829. 33692806 10.3389/fimmu.2021.637829 PMC7937623

[62] Cua DJ , Tato CM . Innate IL‐17‐producing cells: the sentinels of the immune system. Nat Rev Immunol. 2010;10(7):479‐489. 20559326 10.1038/nri2800

[63] Gu C , Wu L , Li X . IL‐17 family: cytokines, receptors and signaling. Cytokine. 2013;64(2):477‐485. 24011563 10.1016/j.cyto.2013.07.022 PMC3867811

[64] Xie S , Li J , Wang JH , et al. IL‐17 activates the canonical NF‐kappaB signaling pathway in autoimmune B cells of BXD2 mice to upregulate the expression of regulators of G‐protein signaling 16. J Immunol. 2010;184(5):2289‐2296. 20139273 10.4049/jimmunol.0903133 PMC2849003

[65] Qian Y , Liu C , Hartupee J , et al. The adaptor Act1 is required for interleukin 17‐dependent signaling associated with autoimmune and inflammatory disease. Nat Immunol. 2007;8(3):247‐256. 17277779 10.1038/ni1439

[66] Chang SH , Park H , Dong C . Act1 adaptor protein is an immediate and essential signaling component of interleukin‐17 receptor. J Biol Chem. 2006;281(47):35603‐35607. 17035243 10.1074/jbc.C600256200

[67] Amatya N , Garg AV , Gaffen SL . IL‐17 signaling: the Yin and the Yang. Trends Immunol. 2017;38(5):310‐322. 28254169 10.1016/j.it.2017.01.006 PMC5411326

[68] Onishi RM , Gaffen SL . Interleukin‐17 and its target genes: mechanisms of interleukin‐17 function in disease. Immunology. 2010;129(3):311‐321. 20409152 10.1111/j.1365-2567.2009.03240.x PMC2826676

[69] Zhang GZ , Liu MQ , Chen HW , et al. NF‐κB signalling pathways in nucleus pulposus cell function and intervertebral disc degeneration. Cell Prolif. 2021;54(7):e13057. 34028920 10.1111/cpr.13057 PMC8249791

[70] Yao Z , Nie L , Zhao Y , et al. Salubrinal suppresses IL‐17‐induced upregulation of MMP‐13 and extracellular matrix degradation through the NF‐kB pathway in human nucleus Pulposus cells. Inflammation. 2016;39(6):1997‐2007. 27590238 10.1007/s10753-016-0435-y

[71] Liu ZC , Wang ZL , Huang CY , et al. Duhuo Jisheng decoction inhibits SDF‐1‐induced inflammation and matrix degradation in human degenerative nucleus pulposus cells in vitro through the CXCR4/NF‐κB pathway. Acta Pharmacol Sin. 2018;39(6):912‐922. 29795361 10.1038/aps.2018.36 PMC6256264

[72] Liu Z , Ma C , Shen J , Wang D , Hao J , Hu Z . SDF‐1/CXCR4 axis induces apoptosis of human degenerative nucleus pulposus cells via the NF‐κB pathway. Mol Med Rep. 2016;14(1):783‐789. 27220474 10.3892/mmr.2016.5341 PMC4918601

[73] Li JK , Nie L , Zhao YP , et al. IL‐17 mediates inflammatory reactions via p38/c‐Fos and JNK/c‐Jun activation in an AP‐1‐dependent manner in human nucleus pulposus cells. J Transl Med. 2016;14:77. 26988982 10.1186/s12967-016-0833-9 PMC4794827

[74] Walsh MC , Lee J , Choi Y . Tumor necrosis factor receptor‐associated factor 6 (TRAF6) regulation of development, function, and homeostasis of the immune system. Immunol Rev. 2015;266(1):72‐92. 26085208 10.1111/imr.12302 PMC4799835

[75] Zepp JA , Liu C , Qian W , et al. Cutting edge: TNF receptor‐associated factor 4 restricts IL‐17‐mediated pathology and signaling processes. J Immunol. 2012;189(1):33‐37. 22649194 10.4049/jimmunol.1200470 PMC3590847

[76] Wu L , Chen X , Zhao J , et al. A novel IL‐17 signaling pathway controlling keratinocyte proliferation and tumorigenesis via the TRAF4‐ERK5 axis. J Exp Med. 2015;212(10):1571‐1587. 26347473 10.1084/jem.20150204 PMC4577838

[77] Zhang F , Zhao X , Shen H , Zhang C . Molecular mechanisms of cell death in intervertebral disc degeneration (review). Int J Mol Med. 2016;37(6):1439‐1448. 27121482 10.3892/ijmm.2016.2573 PMC4866972

[78] Zhang HJ , Liao HY , Bai DY , Wang ZQ , Xie XW . MAPK /ERK signaling pathway: a potential target for the treatment of intervertebral disc degeneration. Biomed Pharmacother. 2021;143:112170. 34536759 10.1016/j.biopha.2021.112170

[79] Shaulian E , Karin M . AP‐1 as a regulator of cell life and death. Nat Cell Biol. 2002;4(5):E131‐E136. 11988758 10.1038/ncb0502-e131

[80] Ye N , Ding Y , Wild C , Shen Q , Zhou J . Small molecule inhibitors targeting activator protein 1 (AP‐1). J Med Chem. 2014;57(16):6930‐6948. 24831826 10.1021/jm5004733 PMC4148154

[81] Yoon HS , Park CM . Chrysoeriol ameliorates COX‐2 expression through NF‐κB, AP‐1 and MAPK regulation via the TLR4/MyD88 signaling pathway in LPS‐stimulated murine macrophages. Exp Ther Med. 2021;22(1):718. 34007327 10.3892/etm.2021.10150 PMC8120564

[82] Xu HG , Cheng JF , Peng HX , et al. JNK phosphorylation promotes natural degeneration of cervical endplate chondrocytes by down‐regulating expression of ANK. Eur Rev Med Pharmacol Sci. 2013;17(17):2335‐2344. 24065227

[83] Waite JC , Skokos D . Th17 response and inflammatory autoimmune diseases. Int J Inflam. 2012;2012:819467. 22229105 10.1155/2012/819467 PMC3249891

[84] Subramaniam SV , Cooper RS , Adunyah SE . Evidence for the involvement of JAK/STAT pathway in the signaling mechanism of interleukin‐17. Biochem Biophys Res Commun. 1999;262(1):14‐19. 10448060 10.1006/bbrc.1999.1156

[85] Huang F , Kao CY , Wachi S , Thai P , Ryu J , Wu R . Requirement for both JAK‐mediated PI3K signaling and ACT1/TRAF6/TAK1‐dependent NF‐kappaB activation by IL‐17A in enhancing cytokine expression in human airway epithelial cells. J Immunol. 2007;179(10):6504‐6513. 17982039 10.4049/jimmunol.179.10.6504

[86] Hu B , Wang J , Wu X , Chen Y , Yuan W , Chen H . Interleukin‐17 upregulates vascular endothelial growth factor by activating the JAK/STAT pathway in nucleus pulposus cells. Joint Bone Spine. 2017;84(3):327‐334. 27426446 10.1016/j.jbspin.2016.05.014

[87] Binch AL , Cole AA , Breakwell LM , et al. Expression and regulation of neurotrophic and angiogenic factors during human intervertebral disc degeneration. Arthritis Res Ther. 2014;16(5):416. 25209447 10.1186/s13075-014-0416-1 PMC4177417

[88] He WS , Zou MX , Yan YG , et al. Interleukin‐17A promotes human disc degeneration by inhibiting autophagy through the activation of the phosphatidylinositol 3‐kinase/Akt/Bcl2 signaling pathway. World Neurosurg. 2020;143:e215‐e223. 32712400 10.1016/j.wneu.2020.07.117

[89] Shen C , Yan J , Jiang LS , Dai LY . Autophagy in rat annulus fibrosus cells: evidence and possible implications. Arthritis Res Ther. 2011;13(4):R132. 21846367 10.1186/ar3443 PMC3239374

[90] Xu K , Chen W , Wang X , et al. Autophagy attenuates the catabolic effect during inflammatory conditions in nucleus pulposus cells, as sustained by NF‐κB and JNK inhibition. Int J Mol Med. 2015;36(3):661‐668. 26165348 10.3892/ijmm.2015.2280 PMC4533778

[91] Yang J , Yang C , Wang Y , et al. Effect of subcutaneous needling on visual analogue scale, IgG and IgM in patients with lumbar disc herniation: study protocol clinical trial (SPIRIT compliant). Medicine (Baltimore). 2020;99(9):e19280. 32118739 10.1097/MD.0000000000019280 PMC7478818

[92] Capossela S , Schläfli P , Bertolo A , et al. Degenerated human intervertebral discs contain autoantibodies against extracellular matrix proteins. Eur Cell Mater. 2014;27:251‐263. discussion 263. 24706108 10.22203/ecm.v027a18

[93] Silva AJ , Ferreira JR , Cunha C , et al. Macrophages down‐regulate gene expression of intervertebral disc degenerative markers under a pro‐inflammatory microenvironment. Front Immunol. 2019;10:1508. 31333653 10.3389/fimmu.2019.01508 PMC6616110

[94] Rajasekaran S , Soundararajan DCR , Nayagam SM , et al. Modic changes are associated with activation of intense inflammatory and host defense response pathways—molecular insights from proteomic analysis of human intervertebral discs. Spine J. 2022;22(1):19‐38. 34303868 10.1016/j.spinee.2021.07.003

[95] Takenaka Y , Kahan A , Amor B . Experimental autoimmune spondylodiscitis in rats. J Rheumatol. 1986;13(2):397‐400. 3487651

[96] Freidin MB , Keser T , Gudelj I , et al. The association between low back pain and composition of IgG glycome. Sci Rep. 2016;6:26815. 27229623 10.1038/srep26815 PMC4882546

[97] Kawakami M , Tamaki T , Matsumoto T , Kuribayashi K , Takenaka T , Shinozaki M . Role of leukocytes in radicular pain secondary to herniated nucleus pulposus. Clin Orthop Relat Res. 2000;376:268‐277. 10.1097/00003086-200007000-00035 10906884

[98] Wang HQ , Samartzis D . Clarifying the nomenclature of intervertebral disc degeneration and displacement: from bench to bedside. Int J Clin Exp Pathol. 2014;7(4):1293‐1298. 24817926 PMC4014210

